# Painless Reduction of Dislocation

**Published:** 1860-10

**Authors:** 


*Painless Reduction of Dislocation,* is said to have been accomplished through the application of compresses saturated with chloroform. B.

